# Perzipierte Ungleichheit und politische Nachfrage: Wie beeinflussen Wahrnehmungsbias und mediale Rahmen die Einstellungen zur Vermögensteuer?

**DOI:** 10.1007/s11577-022-00809-8

**Published:** 2022-02-15

**Authors:** Ursula Dallinger

**Affiliations:** Fachbereich IV, Abteilung Soziologie, Universität Trier, 54286 Trier, Deutschland

**Keywords:** Vermögensteuer, Präferenzformation, Wahrnehmungsbias, Politische Unterstützung, Öffentliche Meinung, Wealth tax, Preference formation, Perception bias, Political support, Public opinion

## Abstract

Nach einer neueren sozialwissenschaftlichen Debatte nehmen die Bürger die Einkommensungleichheit eher ungenau wahr, was auch ihre Akzeptanz verteilungspolitischer Programme beeinflusst. Die Studie, über die der Artikel berichtet, prüft, ob sich das am Beispiel der Vermögensteuer bestätigen lässt. Die Vermögensteuer wurde in Deutschland 1996 ausgesetzt, jedoch ringt die Politik seit Jahren um ihre Wiedereinführung. Vor dem Hintergrund der Debatte um verzerrte Perzeptionen bei der Formation verteilungspolitischer Präferenzen fragt der Beitrag erstens, wie genau die existierende Steuerlast wohlhabender Haushalte durch den Spitzensteuersatz für Einkommen eingeschätzt wird und ob ein Bias Folgen für die Unterstützung einer Vermögensteuer hat. Ausgehend von Ansätzen, die Massenmedien eine wichtige Rolle bei der Formation verteilungspolitischer Präferenzen zuschreiben, wird zweitens der Einfluss medialer Rahmen zur Vermögensteuer auf die Akzeptanz dieses Instruments überprüft. Nach den Daten eines Onlinesurveys wird die Belastung durch die Spitzensteuer eher zu hoch eingeschätzt. Je stärker diese Steuer überschätzt wird, desto geringer ist die politische Unterstützung einer Vermögensteuer. Framingexperimente mit randomisierten Kontroll- und Treatmentgruppen im Rahmen des Surveys bilden aktuelle Diskurse um die Vermögensteuer ab und rekonstruieren positive Frames – Vermögensteuern als investitionsfördernde Steuerreform, als Beitrag zum Abbau von Staatsschulden durch Corona, wie auch ablehnende Frames – Einschränkung von Investitionen und Verlust von Arbeit bei Belastung der Unternehmen. Das Verfügbarmachen möglicher Arbeitsplatzverluste senkt die Unterstützung einer Vermögensteuer höchst signifikant. Die mehrheitlich starke Zustimmung fällt auf die mittlere Kategorie „teils/teils“ ab, ein Signal der Unentschiedenheit. Das Ringen um Benennungsmacht ist also offen. Die Unterstützung einer Vermögensteuer wird unsicher, je mehr politische Kommunikation den Rahmen bedrohter Arbeitsplätze aktiviert.

## Abstract (en)

According to a recent social science debate, citizens tend to perceive income inequality rather inaccurately, which also influences their acceptance of redistributive policy programmes. The study reported in this article examines whether this can be confirmed using the example of the wealth tax. The wealth tax was suspended in Germany in 1996, but politicians have been debating its reintroduction for several years. Against the background of the debate on biased perceptions in the formation of distributional policy preferences, the article asks, first, how accurately the existing tax burden on wealthy households through the top income tax rate is assessed and whether a bias has consequences for the support of a wealth tax. Second, based on approaches that attribute an important role to mass media in the formation of distributional policy preferences, the influence of media framing on the acceptance of this controversial instrument is examined. According to data from an online survey, the burden of the top income tax tends to be overestimated. The more the tax is overestimated, the lower the political support for a wealth tax. Framing experiments with randomized control and treatment groups have mapped current discourses around the wealth tax and reconstructed positive frames—wealth taxes as an investment promoting tax reform, as a contribution to the reduction of national debt caused by the coronavirus pandemic—as well as negative frames—restriction of investments and loss of jobs if companies are burdened. Exposing potential job losses significantly lowers the support for a wealth tax. Strong support drops to the middle category of “partly/partly,” a signal of indecision. The struggle for naming power is thus open. Support for a property tax becomes uncertain the more that political communication activates the framework of threatened jobs.

## Einleitung

Seit einigen Jahren wird in Öffentlichkeit und Sozialwissenschaften diskutiert, wie genau Bürger und Bürgerinnen wirtschaftliche Ungleichheiten wahrnehmen und welche Folgen ein Wahrnehmungsbias für ihre Nachfrage nach umverteilender Steuer- und Sozialpolitik hat. „Die Deutschen wollen viele Einkommen niedriger besteuern und Vermögen umverteilen. Was aber sind ihre Wünsche wert, wenn sie die Wirklichkeit nicht kennen?“ schrieb etwa Die Zeit (Altmann et al. 2018; auch Rudzio 2012). Wenn die Fakten zur Ungleichheitsentwicklung nur vage bekannt seien, könne die Nachfrage der Bürger nach redistributiver Politik kaum sachgerecht sein (Niehues 2014; Engelhardt und Wagener 2014, 2018; Gimpelson und Treisman 2015).

Wie sehr Ungleichheit ungenau perzipiert wird und wie ein Bias die politische Nachfrage beeinflusst, ist allerdings wenig erforscht. Der vorliegende Beitrag analysiert diese Frage am Beispiel der Vermögensteuer. Sie wird, wie die Spitzensteuer für Erwerbseinkommen, die Erbschafts- und die Grundsteuer, als „Reiche“ belastendes Umverteilungsinstrument betrachtet. Untersucht wird, wie weitreichend verzerrte Informationen zu „Reichensteuern“ sind und wie eine möglicherweise ungenaue Perzeption der steuerlichen Belastung Wohlhabender die Unterstützung für eine Vermögensteuer beeinflusst. Wie wirkt sich ein Bias bei der wahrgenommenen Einkommenssteuer auf die Zustimmung zur Vermögensteuer, deren Wiedereinführung seit Jahren diskutiert wird, aus?

Die Vermögensteuer ist seit ihrer Aussetzung Ende 1996 nach einem Urteil des Bundesverfassungsgerichts nicht mehr in Kraft. Befürworter versuchen, sie zu revitalisieren und brachten sie schon im Wahlkampf vor der Bundestagswahl 2013 als Instrument zum Abbau der hohen Staatsverschuldung durch Rettungsprogramme im Zuge der Finanzmarktkrise ein. Für bestimmte politische Kräfte ist eine Vermögensteuer primär wegen der hohen Einkommens- und Vermögenszugewinne an der Spitze der Verteilung wünschenswert (Bach et al. 2016; Bach und Thiemann 2016; DGB Bundesvorstand 2019; Piketty 2020). Opponenten erwarten hingegen geringe Umverteilungseffekte, dafür aber Standortnachteile, hohe Erhebungskosten, Ausweichreaktionen und verfassungsrechtliche Hürden, die bereits zu ihrer Aussetzung führten (Hey et al. 2012; Fuest 2019). Im Jahr 2020 war die Vermögensteuer zum Abbau coronabedingter Staatsschulden im Gespräch.

Zwar besteht in der deutschen Öffentlichkeit Konsens darüber, dass „starke Schultern“ einen größeren Teil der Steuer- und Abgabenlast tragen sollen. Jedoch wird darum gerungen, wie groß diese Last sein darf. Auseinandersetzungen um die Besteuerung Reicher haben nach Scheve und Stasavage (2016) eine typische Konstellation: Mit Einführung des allgemeinen Wahlrechts kann die weniger begüterte Bevölkerungsmehrheit im Grunde „Reichensteuern“ durchsetzen. Empirisch geschehe dies besonders dann, wenn Bilder über besondere Privilegien und Begünstigungen Wohlhabender kursieren, nicht etwa bei hoher Vermögensungleichheit. Allerdings enthält diese Konstellation Vetospieler, die ihren Einfluss gegen eine Besteuerung „Reicher“ zur Geltung bringen. Interessenorganisationen der Unternehmen und der Finanzmärkte^1^ erinnern an „negative Anreize“ – d. h. an die drohende Abwanderung von Unternehmen und das Zurückfahren von Investitionen mit der Folge fehlender Arbeitsplätze. Beide Seiten arbeiten daran, ihre Sicht glaubwürdig zu machen; die eine an der Überlastung der Wirtschaft, die andere an den besonderen Zugewinnen der Vermögenden (Piketty 2015). Ein Kampf um Benennungsmacht findet statt (Bourdieu 1985), in dem Medien die Narrative der diversen politischen und wirtschaftlichen Eliten transportieren (Grusky und McLean 2015) und am Ringen um die „richtige“ Wahrnehmung mitwirken.

Auch in dieser Konstellation rücken Perzeptionen ökonomischer Ungleichheit in den Vordergrund, die schon die Forschung zu Umverteilungspolitik entdeckte, nachdem ein Einfluss der faktischen Ungleichheit auf die verteilungspolitischen Einstellungen der Bürger (Meltzer und Richard 1981) nicht systematisch nachweisbar war. Es mehren sich Hinweise zur vielmehr durch subjektive Ungleichheit beeinflussten politischen Nachfrage (Hochschild 2001; Kenworthy und McCall 2008; Norton und Ariely 2011; OECD 2021). Es ist anzunehmen, dass auch die Medienberichterstattung perzipierte Ungleichheit bestimmt. Somit sind Reichtum und dessen Ursachen auch geglaubte Fakten (Zaller 1992). Medien machen Wissen über Gewinner und Verlierer der wirtschaftlichen Entwicklung über Vor- und Nachteile verteilungspolitischer Instrumente verfügbar und bieten politischen Eliten eine Plattform (Lippmann 1964; Zaller 1992). Wie Medien Urteile über ökonomische Ungleichheit und politische Instrumente, wie die Vermögensteuer, beeinflussen, ist wenig erforscht. Daher fragt der Beitrag, welche „Benennungsmacht“ die mediale politische Kommunikation auf Einstellungen zur Vermögensteuer ausübt.

Die Daten für Analysen sowohl der wahrgenommenen Spitzensteuerbelastung und deren Einfluss auf die Akzeptanz einer wieder eingeführten Vermögensteuer wie auch zum Einfluss medialer Rahmen stammen aus einem Onlinesurvey von 2020, das neben Fragen zur perzipierten Steuerbelastung und Ungleichheit außerdem drei Surveyexperimente (plus Kontrollgruppe) enthält. Das Folgende skizziert zunächst die Forschung über Einstellungen zur Vermögensteuer, zu ungenauen Ungleichheitsperzeptionen und zur Rolle der Medien. Dann wird der Onlinesurvey erläutert. Die Resultate verweisen auf überschätzte Spitzensteuern, die nach multivariaten Analysen die Unterstützung einer Vermögensteuer senken. Im Vergleich zum Effekt des Bias bei Steuerfakten ist allerdings die zu hoch bewertete Ungleichheit in Deutschland deutlich einflussreicher. Framingexperimente zeigen die „Erreichbarkeit“ der Bürger für in den Medien präsente Argumente.

## Stand der Forschung

### Einstellungen zu „Reichensteuern“

Steuern beschaffen Mittel zur Finanzierung öffentlicher Güter, wie Bildung oder Mindestsicherung, oder schließen Lücken in den Sozialversicherungen (z. B. gesetzliche Rentenversicherung). Sie können wirtschaftliche Ungleichheit reduzieren, ihr aber auch Vorschub leisten. Den letztgenannten Effekt hatte die stufenweise Senkung des Spitzensteuersatzes von 53 auf 42 % im Jahr 2004 und die 2006 von 16 auf 19 % erhöhte Mehrwertsteuer (Biewen und Juhasz 2012; Bach et al. 2015; Horn et al. 2017; Binder und Haupt 2018). Die Zugewinne der Wohlhabenden bei Einkommen und Vermögen im Vergleich zu mittleren und unteren Einkommensschichten (Scholtz 2010; Anselmann und Krämer 2012) befeuern öffentliche Debatten um höhere Steuern für „Reiche“ (Erbschafts‑, Vermögens- oder Spitzensteuer).

Die öffentliche Meinung zur Vermögensteuer nahm einen interessanten Verlauf. Im Jahr 2007 stimmten nur 35 % der Bevölkerung einer Vermögensteuer zu (Bertelsmann Stiftung Pressemitteilung 10.12.2007).^2^ Einige Jahre später war die politische Unterstützung stark gestiegen. Forsa ermittelte 2012 in einer Umfrage im Auftrag der NGO Campact, dass 77 % der Befragten eine Vermögensabgabe als gerechte Beteiligung großer Vermögen an den Lasten der Finanzkrise befürworten. Die 2013 von Infratest dimap (2013) durchgeführte Befragung kommt zu 66 % Zustimmung. Im Dezember 2019 fanden dann 72 % der Befragten Vermögensteuern angemessen (ARD Deutschland-Trend).^3^ Nach meinen im November 2020 erhobenen Daten stimmen 66 % einer Vermögensteuer zu (siehe Tab. 2 im Anhang). Die Resultate der Befragungen sind zwar nicht direkt vergleichbar, zeigen aber einen Anstieg der Unterstützung nach 2007 – vermutlich ein Resultat der Politisierung der Vermögensteuer in der Finanz- und Staatsschuldenkrise. Das Zustimmungsniveau schwankt in einer gewissen Bandbreite über die diversen Studien. Da in den eigenen online erhobenen Daten die unterste Bildungsgruppe (mit überdurchschnittlicher Zustimmung zu diesem steuerpolitischen Instrument) etwas unterrepräsentiert ist, wäre die hier gemessene Zustimmung etwas nach oben zu korrigieren.

Was wissen wir aber jenseits der Demoskopie über die Formation politischer Präferenzen gegenüber Vermögensteuern, die wie progressive Steuern, Erbschafts- und Spitzensteuern Reiche be- oder entlasten und die man summarisch als „Reichensteuern“ bezeichnet? Die Besteuerung wohlhabender Haushalte ist ein Thema im Fadenkreuz aus Forderungen nach stärkerer Besteuerung hoher Einkommen und Vermögen in einer Phase von deren wachsender Konzentration einerseits und liberaler Kritik am staatlichen Zugriff auf privaten wirtschaftlichen Wohlstand andererseits.

Die Struktur – wenige Reiche zahlen, die Mehrheit der Wähler profitiert – garantiert keineswegs die Unterstützung der Bevölkerung. In der Schweiz scheiterte ein Referendum zur Einführung einer Erbschaftssteuer, deren Ertrag in bessere Grundrenten fließen sollte, an einer Medienkampagne zu den Lasten für kleine und mittlere Familienunternehmen und wegfallende Arbeitsplätze (Emmenegger und Marx 2018). In den USA erzeugte die Regierung politische Unterstützung für eine Steuerreform, die Reiche deutlich, die Masse der Bürger aber wenig entlastete, indem die Erbschaftssteuer in den Medien als „death-tax“ geframt wurde und man kommunizierte, dass selbst „Durchschnittsbürger“ zahlen müssten (Bartels 2008; Kap. 6). Vignettenstudien gehen Deutungs- und Rechtfertigungsmustern für die Akzeptanz der steuerlichen Belastung Vermögender nach. „Erbschaften als verdientes Vermögen, das in der Familie bleiben muss“ macht den steuerlichen Zugriff auf „die Reichen“ schwierig (Groß und Lang 2018). Je nach den perzipierten Gründen für Reichtum – individuelle Leistung oder privilegierte Herkunft – erscheint Vermögen als verdient oder unverdient mit Folgen für die Akzeptanz einer Vermögensteuer (Sachweh und Eicher 2018; auch Beckert 2017).

Einstellungen zur Progressivität von Steuern seien entscheidend durch Gerechtigkeitsüberzeugungen („beliefs“), weniger durch Eigeninteresse bestimmt (Hennighausen und Heinemann 2014). Für die Rolle von Interessen spricht aber die institutionentheoretische Studie von Berens und Gelepithis (2019): Auf Arme konzentrierte Sozialleistungen mindern die Unterstützung für progressive Steuern, breit über die Sozialschichten gestreute Leistungen steigern sie. Die Mittelschicht favorisiert progressive Steuern nur, wenn sie davon profitiert. Ebenfalls der Forschung zu „comparative welfare attitudes“ verpflichtet, bilden Roosma et al. (2016) individuelle Profile der Legitimität progressiver Steuern.

Allerdings versäumen Studien, die in bestimmten Wertideen die Legitimationsgrundlagen für Vermögensteuern sehen, dass politische Einstellungen kommunikativ vermittelt sind. Zudem abstrahiert die Haltung, wonach Wähler und Wählerinnen die Legitimationsgrundlage der Politik seien, von politischen Eliten und Interessengruppen, die über die Medien ihre Sichtweise vermitteln und durchsetzen wollen (Jacobs und Shapiro 2000; Bartels 2008; Faas und Schön 2014). Die Formation politischer Präferenz sollte dynamischer modelliert und deren Abhängigkeit von (medienvermittelter) politischer Kommunikation berücksichtigt werden. Dafür sprechen auch die oft diffusen Kenntnisse Befragter über die Wirkungsweise des Sozial- und Steuersystems und die vagen Vorstellungen über die Umverteilungseffekte von Steuern (Barnes 2015; Kim et al. 2016).

Scheve und Stasavage bieten ein dynamisches Modell der Sichtweisen der Bürger: „Societies tax the rich when people believe that the state has privileged the wealthy, and so fair compensation demands that the rich be taxed more heavily than the rest“ (Scheve und Stasavage 2016, S. 4). Allerdings gibt es eine Auseinandersetzung um das Bild von den „Reichen“. In Demokratien mit gleichem Wahlrecht kann formal die Mehrheit Reichensteuern auferlegen. Jedoch hat die Wirtschaft strukturelle Macht und gute Durchsetzungschancen, indem sie „adverse incentive effects“ aktiviert (Scheve und Stasavage 2016, S. 12) und die Sorge ins Spiel bringt, dass bei stärkerer Belastung der Wirtschaft und der Vermögen die Investitionen unterbleiben, Firmen abwandern und letztlich Arbeitsplätze fehlen. Die Wirtschaft kann in öffentlichen Debatten um Steuern für Wohlhabende als Vetospieler auftreten, da die strukturelle Macht der Wirtschaft für abhängig Beschäftigte kaum zu negieren ist.^4^ Interessenorganisationen der „Wohlhabenden“ erinnern in den Medien an mögliche adverse Effekte, während Organisationen weniger Einkommensstarker bestrebt sind, die Privilegierung „der Reichen“ sichtbar zu machen (Köppe et al. 2007; Smith Ochoa 2019). Wie nachhaltig Medien die Präferenzen zur Vermögensteuer beeinflussen, wird später mit Framingexperimenten geprüft.

### Perzeptionen von Ungleichheit

Da nicht schon die faktische Ungleichheit Forderungen nach einer korrigierenden Sozial- und Steuerpolitik auslöst, interessiert sich die Forschung zunehmend für die Perzeption wirtschaftlicher Ungleichheit. Diese ist in vielen Bereichen ungenau. So stimmen weder die geschätzte und die objektive Armutsquote (Gimpelson und Treisman 2015), noch die geschätzte und die faktische Arbeitslosenquote (Niehues 2019) überein. Deutsche glauben, die meisten lebten in den unteren Einkommensdezilen nahe oder unter der Armutsschwelle, obwohl die Mehrheit der Deutschen faktisch der Mitte zuzuordnen ist (Niehues 2014). Bürger der Niederlande oder der Schweiz stufen die Einkommensverteilung ihrer Länder hingegen realistisch als Mittelschichtgesellschaft ein. Ungleichheit wird aber nicht durchgängig gravierender perzipiert als sie faktisch ist. Die subjektive Wahrnehmung glättet häufig Diskrepanzen: So etwa, wenn die Löhne einfacher Berufe höher eingeschätzt werden als sie faktisch sind (Kelley und Evans 1993).^5^ Auch scheinen sich subjektive Urteile über Ungleichheit im Zeitverlauf an die gestiegene Ungleichheit anzupassen (Schröder 2017), was auch experimentell gezeigt wurde: Das Erleben von Ungleichheit im Experiment förderte affirmative Einstellung zu Ungleichheit (Trump und White 2018), was gestützt auf die Sozialpsychologie als Wunsch zur Systemrechtfertigung erklärt wird.

Die Konzentration der Vermögen am oberen Ende – ein in Urteile über eine Vermögensteuer vermutlich einfließender Aspekt – wird unterschätzt (Bartels 2008; Norton und Ariely 2011). Auch die perzipierte Steuerbelastung mag relevant sein. So gibt es Hinweise, dass die von Haushalten mit geringen Einkommen gezahlten Steuern überschätzt, hingegen die von Haushalten mit hohen Einkommen gezahlten Steuern unterschätzt werden (Niehues 2019).^6^

Insgesamt sind Diagnosen zu Ungleichheitsperzeptionen sehr uneinheitlich. Es gibt sowohl Befunde zu „überspitzt“ wahrgenommener (Hüther und Diermeyer 2019; Niehues 2014, 2016) als auch zu „verharmloster“ Ungleichheit. Zu prüfen sind daher die Effekte übersteigert perzipierter wie auch zu gering eingeschätzter Ungleichheit auf die politischen Präferenzen oder die gewünschte Verteilungspolitik. In Bezug auf die Besteuerung der „Reichen“ ist also zu fragen, wie ungenau das subjektive Wissen der Bürger über die bereits bestehende steuerliche Belastung der Spitzeneinkommen ist oder in welchem Maße Spitzensteuern über- oder unterschätzt werden und ob Verzerrungen Folgen für die politische Unterstützung der Vermögensteuer haben.

#### H1

Je mehr die Belastung der Reichen durch die Einkommenssteuer überschätzt wird, desto geringer ist die Unterstützung der Vermögensteuer. Und umgekehrt: Wird die steuerliche Belastung unterschätzt, steigt die Zustimmung zu einer Vermögensteuer.

Weitere Aspekte perzipierter Ungleichheit wie die Wahrnehmung der eigenen Einkommensposition und der gesellschaftlichen Ungleichheit dürften die Unterstützung einer Steuer für Vermögende beeinflussen. Ein Bias bei der Selbsteinstufung geschieht durch die Einstufung in der Mitte auch von Personen, die kein Mittelschichteinkommen oder keinen Mittelschichtstatus haben. Befragte aus Dezilen unterhalb der Mitte stufen die eigene Einkommensposition höher, Befragte der Dezile oberhalb der Mitte jedoch weiter unten ein als es ihrer Einkommensposition entspricht. Dies lässt Einkommensdiskrepanzen subjektiv schrumpfen, eine Form des gesellschaftlichen Kitts (Wegener 1987). Die aktuelle ökonomische Forschung betrachtet dieses Stauchen der Abstände lediglich als Informationsdefizit, das durch „Aufklärung“ zu beheben sei, was die Präferenzen in Bezug auf Umverteilungspolitik realistischer gestalte und zudem die Steuer- und Abgabebereitschaft der Bezieher hoher Einkommen senkt (Engelhardt und Wagener 2018; Bublitz 2016). Die vorliegende Studie informiert Befragte nicht über ihre Fehleinschätzungen, sondern fasst diese als Teil der sozialen Realität. Sie testet lediglich, wie sehr die Selbsteinstufung (Operationalisierung siehe Abschn. 3) die verteilungspolitischen Präferenzen beeinflusst.

#### H2a

Je höher Befragte ihre eigene Stellung in der Einkommenshierarchie einschätzen, desto eher lehnen sie eine Vermögensteuer ab.

Anders als die Selbsteinstufung ist das Urteil über eine zu ungleiche Gesamtgesellschaft nicht unbedingt an eigene Erfahrungen gekoppelt und eher auf Informationen durch die Medien angewiesen. Jedoch prägt es nach bisherigem Wissen maßgeblicher die politischen Präferenzen als die Einstufung der eigenen Position (Mutz 1998). Während man die Sicht der eigenen Position gewissermaßen aufwertet (durch Selbsteinstufung in der Mitte, siehe oben), fällt das Urteil über die Gesellschaft „kritischer“ oder negativer aus im Verhältnis zur recht großen Zufriedenheit mit der eigenen Lage (Brenke und Kritikos 2017). Daher interessiert, wie sehr die Unterstützung für redistributive Politik in Form von Vermögensteuern durch die wahrgenommene gesellschaftliche Ungleichheit geprägt wird.

#### H2b

Je stärker die Perzeption ausgeprägt ist, die Gesellschaft sei zu ungleich, desto eher wird die Besteuerung des Vermögens unterstützt.

### Medien und Perzeption der Ungleichheit

Erstaunlich selten wird die Medienberichterstattung als Ursache für verzerrt perzipierte wirtschaftliche Ungleichheit untersucht.^7^ Allerdings ist gerade die Einschätzung gesellschaftlicher Ungleichheit medienvermittelt (Happer und Philo 2013; McCall 2013; Petring 2016). Auch die Präferenzen für Vermögensteuern können durch die mediale Berichterstattung beeinflusst werden. Die Medienforschung unterscheidet Priming, Agenda-Setting und Framing als Mechanismen, über die auch die verteilungspolitischen Einstellungen der Bürger erreicht werden können. Bei Priming und Agenda-Setting werden durch unbewusste Informationsverarbeitung erzielte Effekte angenommen. Framing hingegen basiert auf bewusst verarbeiteten Argumenten aus den Medien, die – meist experimentell – zugänglich gemacht werden (Entman 2007).

Für den Agenda*-*Setting-Ansatz entscheidet schon die Häufigkeit des Auftauchens eines Themas in den Medien über seinen Einfluss. Was oft in den Medien ist, ist für die Meinungsbildung verfügbar (Zaller 1992; Maurer 2010), wird vom Publikum als salient betrachtet und strahlt auf Urteile über die Performanz von Parteien und Politikern ab. Agenda-Setting-Effekte werden vermutet, wenn politische Einstellungen und Bewertungen von Parteipolitik mit der Intensität der Medienberichterstattung über das Thema schwanken.

In der Forschung zu ungleichheits- und sozialpolitikbezogenen Einstellungen findet man nur wenige Studien zu Priming und Agenda-Setting. Gilens (1996, 1999) zeigte, dass die US-Medien die afroamerikanische Bevölkerungsgruppe überproportional häufig als Bezieher von Sozialleistungen präsentieren und dass dies die Unterstützung für Sozialausgaben in der Bevölkerung senkt. Seine Studien gelten als Beispiel für Priming, da die unbewusste Wirkung der in den Medien gezeigten Bilder von Schwarzen (negative Attribuierung) analysiert wird.

Agenda-Setting-Effekten gehen Diermeier et al. (2017) mit Daten zur Häufigkeit der Medienberichterstattung einerseits und Einstellungsdaten aus dem Sozio-oekonomischen Panel (SOEP) andererseits nach. Eine intensivere Berichterstattung über Ungleichheit vor dem Interview geht einher mit mehr Befragten, die mangelnde Gerechtigkeit in Deutschland kritisieren. Der Effekt tritt umso eher ein, je mehr Tage berichtet wurde. Allerdings liegen dem Beitrag zwei abhängige Variablen zugrunde. Gerechtigkeitsperzeptionen, die das SOEP bis dahin in einer Welle erfasste, und die Zufriedenheit mit der Wirtschaftslage in Deutschland, für die Daten zwischen 2001 und 2015 vorliegen. Langfristige Paneldaten, die individuelle Variation bei der Perzeption mit variierender Berichtsintensität erklären können, kommen also nur für die Einschätzung der Wirtschaftslage zum Tragen und mit nur eingeschränktem Zeithorizont für Gerechtigkeitsperzeptionen. Die Medienberichterstattung wird über Sekundärdaten, die die Häufigkeit der Berichte zu Ungleichheit auszählen, zugespielt.^8^ Die Richtung oder der Tenor der Berichterstattung bleibt völlig offen, obwohl mit sehr unterschiedlichem Tonfall über Ungleichheit berichtet wird. Zudem bleibt offen, welche dieser Medieninhalte die Befragten tatsächlich rezipierten, ein typisches Defizit der Agenda-Setting-Forschung. Analysen von Medieneffekten im Sinne von Agenda-Setting nehmen hingegen an, dass die Häufigkeit der Themen, die in den Medien auftauchen und die Medienumwelt der Rezipienten bilden, eine Einstellungsänderung auslöst.

Bei der in der vorliegenden Studie untersuchten Vermögensteuer liegen zwar Agenda-Setting-Effekte als Erklärung ihrer über die Zeit steigenden Akzeptanz nahe. Jedoch möchte die Studie direkt den Einfluss bestimmter aktueller Medieninhalte zur Vermögensteuer auf das Publikum prüfen. Die Rezeption spezifischer Argumente und deren Effekte lässt sich am ehesten experimentell durch Framingstudien nachbilden, die kontrollieren, dass bestimmte Medieninhalte tatsächlich rezipiert wurden (siehe Abschn. 3 zu Methoden). Zur Analyse des Einflusses der unterschiedlichen Berichtintensität über Reichtum, Ungleichheit oder steuerpolitische Instrumente auf die Einstellungen der Bürger wäre eine Medieninhaltsanalyse nötig, die nicht nur auszählt, wie häufig das Thema Vermögensungleichheit auftaucht, sondern auch Valenzen^9^ und Narrative in der Berichterstattung ermittelt. Dieser zusätzliche eigene Schritt hätte das Volumen dieses Zeitschriftenbeitrags gesprengt. Da primär die Medieneffekte spezifischer Argumente der politischen Kommunikation relevant sind, werden experimentelle Framinganalysen mit Kontrolle der Rezeption bevorzugt.

Auch in die Analyse zum Einfluss des Bias auf die Akzeptanz der Vermögenssteuer geht die faktische Mediennutzung ein, allerdings nur in Form der Information dazu, wie intensiv Befragte Medien nutzen, um sich über Politik zu informieren. Dazu wird keine gerichtete Hypothese formuliert, da intensive politikbezogene Mediennutzung positive wie negative Effekte haben kann (Bartels 2008).

Frames bieten spezifische Darstellungsweisen eines Problems, selegieren Informationen, formulieren Argumente und machen sie aktuell im Experiment verfügbar. Der Konsum von Medien ist kontrolliert und Auswirkungen der Frames auf die Einstellungen und Urteile über politische Themen lassen sich messen (Zaller 1992; Druckman 2005; Chong und Druckman 2007). Frames werden meist empirischen Mediendebatten entnommen (Kangas et al. 2013; Görres et al. 2019). Ein Framingeffekt liegt vor, wenn sich politische Einstellungen unter dem Eindruck bestimmter Medieninhalte ändern. Er lässt sich den experimentellen Treatments kausal zuordnen, da Frames randomisierten Gruppen mit gleich verteilten Drittvariablen vorgelegt werden. Da Frames akzeptiert oder abgelehnt werden können, sieht man, welche Argumente wirken. Durch differenzielle Analysen lassen sich eventuelle Unterschiede der Framingeffekte bei bestimmten sozialen Gruppen (z. B. nach Bildung, Parteiidentifikation) prüfen.

Kritisiert wird, dass Framingeffekte meist in einmaligen Experimenten festgestellt werden, die Einstellungsänderung aber nicht stabil sein muss. Wiederholte Einstellungsmessungen nach dem Experiment zeigen aber, dass der anfängliche Framingeffekt verschwindet (de Vreese 2004; Maurer 2010; Matthes und Schemer 2012; Schemer 2014; Schmidt-Catran und Czymara 2020). Diese Kritik greift jedoch nicht, wenn Themen wiederholt in den Medien präsent sind. Die politische Kommunikation über „Arm und Reich“ kennzeichnet, dass Fakten und Argumente wiederholt werden. Politische Akteure selbst wollen durch das Wiederholen bestimmter Narrative eine für ihre Positionen günstige Medienumwelt fördern. Auch in der Debatte um die Vermögensteuern sind Rahmen bereits eingeschliffen, da schon nach der Finanzmarkt- und Staatsschuldenkrise 2012 Bündnis90/Die Grünen und Die Linke Gesetzesentwürfe zu einer Vermögensabgabe einbrachten und zudem angesichts der Einkommens- und Vermögenszugewinne der Reichen die linksorientierten Parteien immer wieder eine stärkere steuerliche Belastung Wohlhabender fordern. Die Framingexperimente zur Vermögensteuer der vorliegenden Studie operieren also in einem vorgeprägten Raum. Die bereits hohe Zustimmung zur Vermögensteuer (siehe 2.1) dürfte durch positive Rahmen kaum weiter steigen. Aber die Experimente können zeigen, wie das zentrale Argument des Vetospielers – eine Steuer auf Vermögen bedeute negative Anreize für die Wirtschaft und Arbeitsplatzverluste – auf Befragte wirken.

#### H3

Das Experiment, das negative Effekte einer Vermögensteuer auf Arbeitsplätze aktuell verfügbar und salient macht, lässt die Zustimmung zu diesem politischen Instrument sinken.

### Debatten um Vermögensteuern in Deutschland

Die Vermögensteuer ist seit Jahren medial präsent. Schon nach der Finanz- und Staatsschuldenkrise von 2008/2009 wurden im Vorfeld der Bundestagswahl 2013 vermögensbezogene Steuern gefordert. Die Linke^10^ wie auch Bündnis 90/Die Grünen^11^ brachten 2012 ihre Gesetzesentwürfe ein. Die SPD platzierte sich nicht eindeutig, obwohl sie sich kontinuierlich zugunsten einer Vermögensteuer geäußert und ein Gutachten beauftragt hatte (Bach et al. 2016). Dieses stellt hohe Steuereinnahmen und geringe Erhebungskosten in Aussicht, was Kritiker einer Vermögensteuer infrage stellen. Neben den Parteien beteiligten sich seit 2012 der Paritätische Gesamtverband,^12^ die Gewerkschaften^13^ und Verdi^14^ an der Debatte; unter anderem, indem sie Repräsentativbefragungen zu Einstellungen der Bevölkerung zur Vermögensteuer beauftragten und in den Medien vorstellten. Die Kritik reagierte auf die Gesetzesentwürfe mit einem Gutachten (Hey et al. 2012), das Reichensteuern aus juristischer und ökonomischer Sicht negativ bewertete und Probleme bei der Feststellung von Vermögen wie auch negative wirtschaftliche Effekte unterstrich.

Auch im Rahmen der Bundestagswahl 2017 profilierten sich die Parteien mit Steuerthemen. Die CDU lehnte Steuererhöhungen ab, die Mittelstand und Facharbeiter träfen, und sieht wegen der geringen Verschuldung und Einhaltung des 3 %-Kriteriums der Europäischen Union, einer starken Wirtschaft und solider Haushaltspolitik wenig Anlass für Vermögensteuern. Bündnis 90/Die Grünen sprachen sich für einen höheren Beitrag Vermögender zum Gemeinwesen aus, mithin für Vermögensteuern. Die SPD plante „Reichensteuern“, konkret einen Aufschlag von 3 % auf den Spitzensteuersatz und eine Erbschaftssteuerreform, aber keine Vermögensteuer. In den Sondierungsgesprächen zur Großen Koalition (GroKo) nach der Wahl und nachdem eine Jamaika-Koalition aus FDP, Bündnis 90/Die Grünen und CDU scheiterte, wurde Anfang 2018 zunächst eine auf 45 % erhöhte Spitzensteuer verhandelt, um höhere Einkommen an öffentlichen Ausgaben zu beteiligen. Die Pläne zur Spitzensteuer ließen sich nicht gegen den größeren Koalitionspartner durchsetzen. Die Medien wägten anschließend ab, ob die erzielten Kompromisse bezüglich der stärkeren Belastung Wohlhabender – fortbestehender Solidaritätszuschlag für Reiche und Grundsteuerreform – ausreichen. Als im November 2018 eine unzufriedene SPD mit dem Ende der GroKo drohte und sich der Vermögensteuer zuwandte, wurde die Berichterstattung intensiver. Proteste der „Gelbwesten“ im Jahr 2018 in Frankreich, ausgelöst durch die Abschaffung der Vermögens- und Reichensteuer und die Einführung einer Benzinsteuer, schufen die Aufmerksamkeit auch deutscher Medien für „Reichensteuern“.

Im Laufe des Jahres 2019 intensivierte sich die Kontroverse um die Vermögensteuer. Befürworter verwiesen auf die „aufgehende Schere zwischen Arm und Reich“ sowohl bei Einkommen als auch Vermögen. Kritiker betonten zu erwartende Schäden für kleine und mittlere Unternehmen, die kaum auf in Betrieben gebundenes Vermögen Steuer zahlen können. Erneut wurden die negativen ökonomischen Effekte einer Vermögensteuer angeführt (Kieler Weltwirtschaftsinstitut) in einer Studie im Auftrag des „Haus- und Grundbesitzerverbandes“; Bundesvereinigung der deutschen Arbeitgeberverbände (https://arbeitgeber.de/newsroom/publikationen/) und eine dauerhafte Abgabe mit Verweis auf Artikel 14 des Grundgesetzes als verfassungswidrig bezeichnet. Zudem sei eine Vermögensteuer wegen der guten Schuldentragfähigkeit Deutschlands unnötig (IFO-Schnelldienst 2020).

Der im August 2019 der Öffentlichkeit präsentierte Präsidiumsbeschluss der SPD zur Vermögensteuer^15^ ließ die Presse reagieren. Der Beschluss der SPD zur Vermögensteuer auf dem Parteitag im Dezember 2019^16^ intensivierte die Mediendebatte bis in die ersten Monate des Jahres 2020 hinein. Seit der ab März 2020 um sich greifenden Coronapandemie wird die Vermögensteuer nun als einmalige Vermögensabgabe gerahmt (wie schon 2012/13), die Bezieher sehr hoher Einkommen und Vermögen an der Staatsschuldenfinanzierung beteiligt. Während Befürworter vor der Coronakrise die Konzentration der Vermögen und Gerechtigkeitsargumente anführten (Beschluss SPD Bundesparteitag),^17^ werden seit 2020 nur noch die Beteiligung am Schuldenabbau und an Zukunftsinvestitionen in Digitalisierung, Infrastruktur und Gesundheitswesen als Argumente präsentiert. Bündnis 90/Die Grünen äußerten sich bei ihrem Bundesparteitag Ende 2020 nicht mehr zugunsten der Einführung einer Vermögensteuer, sondern blieben vage. Die Linke, die Vermögensteuern kontinuierlich aus Gerechtigkeitserwägungen fordert, beauftragte erneut ein Gutachten (Bach 2020), über das die Tagesthemen am 03.11.2020 ausführlich berichten. CDU/CSU und FDP lehnen neue Steuern ab, die die Wirtschaft in der Krise zusätzlich belasteten.^18^

## Methoden und Daten

Die Studie beruht auf einem im November 2020 durchgeführten Onlinesurvey mit einem Sample von 1300 Befragten. Es handelt sich nicht um eine Zufallsstichprobe auf der Basis probabilistischer Verfahren, sondern um ein Quotensample für die deutsche Bevölkerung zwischen 18 und 75 Jahren. Die Selektion der Befragten nach Quoten für die Merkmale Alter, schulische Bildung, Haushaltseinkommen und Erwerbsstatus erzielt jedoch eine Annäherung der Verteilung soziodemografischer Merkmale an die Verteilung in der Bevölkerung. Nach Tab. 3 im Anhang ist dies weitgehend gelungen. Allerdings enthält das Onlinesample der Studie weniger Befragte mit oder ohne Hauptschulabschluss als Destatis nach der amtlichen Statistik mit Mikrozensusdaten ausweist. Die zu geringe Repräsentation dieser Gruppe findet sich auch im ALLBUS oder ESS (European Social Survey). Um die Verteilung der Nettohaushaltseinkommen im Onlinesample an Referenzdaten zu prüfen, ist Destatis wegen der zu unterschiedlichen Einkommensgruppen nicht geeignet. Aber ALLBUS und European Social Survey bieten sich aufgrund ähnlicher Kategorien an. Die Verteilung der Nettohaushaltseinkommen im ALLBUS und dem hier zugrundeliegenden Sample ist vergleichbar, während im ESS Haushalte mit höheren Einkommen (4000–5000 €, 5000 € und mehr) stärker vertreten sind. Erwähnt sei noch der hohe Anteil an Erwerbstätigen in der eigenen Onlinebefragung. Mit Onlinebefragungen werden tendenziell nicht Erwerbstätige erreicht, wenn nicht wie hier durch Quotierung gegengesteuert wird. Die Analysen verwenden eine Gewichtungsvariable, die Abweichungen bei Bildungsniveau und Nettohaushaltseinkommen korrigiert. Obgleich dies noch kein repräsentatives Sample erzielt, sind strukturtreue Aussagen über die theoretisch relevanten Zusammenhänge möglich. Repräsentativ gültige Aussagen zur Gesamtbevölkerung sind nicht angestrebt.

Bei den nach den Surveyfragen zu Perzeption von Steuern und Ungleichheit platzierten drei Framingexperimenten (plus Kontrollgruppe) ist die Randomisierung gelungen. Die soziodemografischen Merkmale sind in allen Gruppen ähnlich verteilt (siehe Tab. 4 im Anhang).

Die abhängige Variable, die Unterstützung für eine Vermögensteuer, wird mit einer Likert-Skala mit folgendem Wortlaut erfasst: „Wie sehr unterstützen Sie eine Steuer auf Vermögen ab 1 Mio. €?“. Die Antwortvorgaben lauten: „lehne voll und ganz ab“, „lehne ab“, „teils/teils“, „stimme zu“, „stimme voll zu“.

Die Perzeption der existierenden Spitzensteuer wird in zwei Schritten erfasst. Zunächst werden Befragte aufgefordert, den Prozentsatz subjektiv und spontan zu schätzen. Betont wird, dass keine korrekte Angabe erwartet wird. Dann wird die Differenz zwischen geschätzter und faktischer Spitzensteuer (momentan 42 %) ermittelt und der Umfang an Fehleinschätzungen der Einkommenssteuer grob klassifiziert, um Abweichung von der faktischen Spitzensteuer nach oben und unten zu erfassen. Dabei wird ein Toleranzspielraum von bis zu 3 % um den faktischen Wert akzeptiert. So gelten Schätzungen in der Spanne zwischen 39 % und 45 % als korrekt. Liegen sie über 45 %, sind sie nach oben verzerrt, unter 39 % sind Schätzungen nach unten verzerrt.^19^ Multivariate Analysen stützen sich jedoch auf eine kontinuierliche Variable zum Grad der Fehleinschätzung als individuelle Differenz zwischen geschätztem und faktischem Steuersatz. Positive Werte eines Befragten stehen für überschätzte Steuerbelastung (z. B. wenn 60 % geschätzt wurde, ist die Differenz 18), negative Werte verweisen auf eine unterschätzte Steuerbelastung (z. B. wenn 30 % geschätzt wurde, ergibt sich −12). Je höher der Wert, desto mehr überschätzen Befragte die Steuerlast hoher Einkommen. Der Durchschnitt der Variable liegt bei 4,3, aber die Standardabweichung von 13,3 dokumentiert große Abweichungen nach oben und unten.

Ein zweiter Indikator für Perzeptionen, die verteilungspolitische Präferenzen beeinflussen, ist die geschätzte eigene Position in der Einkommenshierarchie. Die Frage lautet: „Was glauben Sie, wieviel Prozent der Bürger ein geringeres Einkommen als Sie selbst haben?“. Befragte konnten einen Schieberegler auf einer Skala von 0–100 auf die geschätzte Position schieben. Das ergibt ein numerisches Maß dazu, wie weit oben (hohe Zahl) oder unten (niedrige Zahl) man sich in der Einkommenshierarchie ansiedelt. Diese Einschätzung korreliert zwar höchst signifikant mit dem Haushalteinkommen, misst aber wegen des typischen Bias bei der Selbsteinstufung (Tendenz zur Mitte; Wegener 1987) nicht das Gleiche. Problematische Multikollinearität tritt in der Regression nicht auf, da die VIF-Werte der betroffenen Variablen bei 1,3 liegen.

Die Bewertung der gesellschaftlichen Ungleichheit wird erfasst mit dem Item „Wie beurteilen Sie die folgende Aussage? Die Einkommensunterschiede in Deutschland sind zu groß“. Die Antwortskala reicht von 1 („stimme überhaupt nicht zu“) bis 5 („stimme voll und ganz zu“) mit den üblichen Abstufungen einer Likert-Skala.

Als Kontrollvariablen gehen Geschlecht, Bildung (mit/ohne Hauptschulabschluss, Realschule, Abitur, Fachhochschulreife = Referenzgruppe), Haushaltseinkommen (in fünf Gruppen) und Intensität der Medienrezeption ein. Das letzte Item wurde mit Informationen zur Quelle der für politische Informationen genutzten Medien^20^ wie auch zur *Häufigkeit* der Mediennutzung gebildet^21^ und ist ein dichotomer Indikator für intensive Mediennutzung (je Medium täglich oder mehrmals pro Woche) gleich ob Fernsehen, Zeitungen und/oder Internet.

Die Experimente entsprechen einem „post-test only-control group design“. Das heißt, die Effekte der Frames werden durch den Vergleich der abhängigen Variablen in Experimental- und Kontrollgruppen festgestellt. Die Frames orientieren sich an den unterschiedlichen Argumentationsweisen in einer Vielzahl gesichteter Artikel in Qualitätszeitungen (FAZ, Die Welt, Süddeutsche Zeitung) in der Zeit vor der Studie (01.01.2020–04.11.2020, Start des Surveys).^22^ Häufige Argumente werden zu typischen Rahmungen zur Frage der Revitalisierung einer Vermögensteuer verdichtet. Es handelt sich also um empirische, nicht um konstruierte Frames, und es handelt sich um häufige Frames. Zusätzlich exakt deren Quantität auszuzählen, hätte nur einen Mehrwert, wenn man dies im Experiment umsetzen könnte. Da politische Kommunikation die Kernargumente wiederholt, ist von einer hohen Frequenz der selegierten Medienhalte auszugehen. Ein Frame thematisiert die Staatsverschuldung durch die Coronakrise als Rechtfertigung für eine „Beteiligung Vermögender an der Schuldenlast“ (Coronaframe). Ein zweiter Frame enthält, dass „eine Vermögensteuer die Kräfte nicht belasten dürfe, die Arbeitsplätze schaffen“ (Arbeitsplatzframe). Ein dritter Rahmen bezeichnet die Vermögensteuer als eine „wachstumsorientierte Steuerreform, die nötige Zukunftsinvestitionen in Technologie, Infrastruktur und das Gesundheitswesen ermögliche“. Die Wortwahl ist eng an die der Printmedien angelehnt. Da nur im Jahr 2020 häufig in den Medien präsente Argumente umgesetzt wurden, fehlt ein Frame zu Gerechtigkeit, obwohl dieser Aspekt zur Debatte um Vermögensteuern zählt. Im Untersuchungsjahr war er wenig präsent und somit für Befragte kaum verfügbar (Zaller 1992).

## Resultate

### Ungenaue Perzeptionen und politische Unterstützung

Hat die perzipierte steuerliche Belastung hoher Einkommen einen Einfluss darauf, ob Befragte eine weitere Besteuerung der Wohlhabenden durch eine Vermögensteuer unterstützen? Um diese zentrale Frage des Beitrags zu analysieren, zeige ich zunächst, wie hoch Befragte den Spitzensteuersatz auf Einkommen schätzen und in welche Richtung ein Bias auftritt. Im Durchschnitt rangiert die Spitzensteuer aus Sicht der Bürger bei 47 %, was den seit 2002 bei 42 % festgelegten Satz überschätzt.

**Figure 1:**
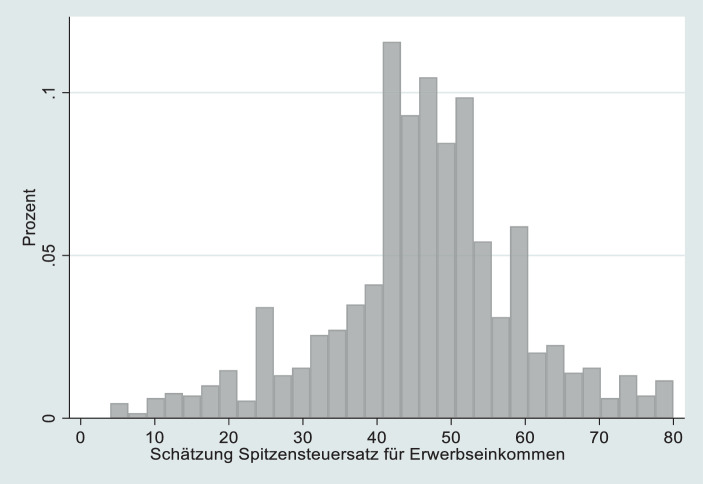

Abbildung 1 betrachtet die Schätzungen detaillierter. Die meisten haben ein recht genaues Wissen und schätzen die Steuer im Bereich zwischen 40 und 45 %. Allerdings ist zu berücksichtigen, dass die Teilnehmer eventuell das Internet konsultierten, um sich zu informieren. Inkorrekte Schätzungen dürfte also verbreiteter sein als hier abgebildet.^23^ Wenn ein Bias vorliegt, dann weicht die wahrgenommene Spitzensteuerbelastung öfter nach oben ab als nach unten. Anders formuliert: Bürger gehen öfter von einer höheren Einkommensteuerbelastung der Spitzenverdiener aus als dies faktisch zutrifft. Korrekte Schätzungen machen 26 % der Befragten, zu gering schätzen 20 % der Befragten die Spitzensteuer und 55 % überschätzen die Spitzensteuerbelastung. Wie ein höherer Anteil der unteren Bildungsgruppe (mit/ohne Hauptschule) im Sample der Onlinebefragung sich auf den Anteil an Personen, die die Spitzensteuer unterschätzen, ausgewirkt hätte, ist schwer zu sagen. Zwar sollten Menschen mit geringer Schulbildung eher urteilen, dass Reiche (zu) wenig Steuern zahlen. Allerdings ist fraglich, ob eine solche Zuschreibung zutrifft, da (mangelnde) Informiertheit oder Bilder über die „Reichen“ intervenieren. Empirisch weisen die Daten keine Beziehung zwischen Schulbildung und Fehleinschätzungen der Spitzensteuer nach. Allerdings sollte künftige Forschung klären, ob alle Bildungsschichten ähnlich fehlinformiert sind.

Die zentrale Frage ist nun, ob die Fehleinschätzung des Spitzensteuersatzes auch die Zustimmung zu Vermögensteuern beeinflusst? Die Resultate der OLS-Regression in Tab. 1, Modell 1 geben Aufschluss darüber. Im Basismodell mit wenigen sozioökonomischen Kontrollvariablen (Schulbildung, Haushaltseinkommen, Geschlecht) erzielt der Grad der Fehleinschätzung einen höchst signifikanten negativen Koeffizienten: Da positive Werte die überschätzte Spitzensteuer erfassen, gilt: Je eher hohe Einkommen schon durch die Spitzensteuer belastet wahrgenommen werden, desto weniger wird eine Vermögensteuer unterstützt. Der Beitrag zur Varianzaufklärung ist mit 3,4 % jedoch klein. Auch die durchschnittlichen Marginaleffekte, dokumentiert in Abb. 3, bestätigen den geringen Effekt. Um den substanziellen Effekt besser einschätzen zu können, ist die Einheit, mit der Fehleinschätzungen gemessen wurden, zu berücksichtigen. Wird die Fehleinschätzung um eine Einheit größer, sinkt die Zustimmung zur Vermögensteuer um den Faktor 0,013. Fehleinschätzungen weichen in der Regel nicht nur um einen, sondern mehrere Prozentpunkte ab (siehe Abb. 1): Ein Irrtum um 10 % nach oben reduziert die Zustimmung um 0,13 Skalenpunkte. Dennoch ist der Effekt klein. Demnach ist *H 1* zwar bestätigt, die substanzielle Wirkung der verzerrten Faktenschätzung aber begrenzt.

**Table 1:** 

	Modell 1	Modell 2	Modell 3	Modell 4
*Fehleinschätzung der Spitzensteuer*	−0,013*** (4,97)	−0,012*** (4,98)	−0,010*** (3,96)	−0,010*** (3,97)
*Ungleichheit zu groß*	–	0,379*** (10,0)	0,367*** (9,91)	0,352*** (9,41)
*Perzeption eigene Position (Skala 1–100)*	–	–	−0,007*** (4,02)	−0,007*** (3,87)
*Mediennutzung Politik hoch*	–	–	–	0,360*** (5,20)
*Bildung (Ref. Abi, FHreife)*				
Hauptschulabschluss	0,264* (2,68)	0,173^**t**^ (1,82)	0,149 (1,58)	0,137 (1,46)
Realschule	0,261** (3,14)	0,200* (2,50)	0,152* (1,89)	0,125 (1,57)
*Geschlecht weiblich*	−0,058 (0,82)	−0,070 (1,02)	−0,095 (1,40)	−0,055 (0,81)
*Haushaltseinkommen*	−0,105** (−3,34)	−0,037 (−1,19)	0,007 (0,21)	−0,008 (0,24)
*Konstante*	4,06 (0,132)***	2,30 (0,218)***	2,58 (0,228)***	2,47 (0,227)***
*R‑Sq*	3,9 %	11,1 %	12,1 %	13,9 %

Gezeigt werden unstandardisierte Koeffizienten. In Klammern t‑statistik; bei Konstante Standardfehler. Daten gewichtet. *N* = 1237

*p < 0,05, **p < 0,01, ***p < 0,001

Im Vergleich zur Fehlperzeption der Spitzensteuer ist die Wahrnehmung der Ungleichheit entscheidender für die Unterstützung der Vermögensteuer (Modell 2). Wird gesellschaftliche Ungleichheit als zu groß bewertet, steigt der Wunsch nach einer Vermögensteuer höchst signifikant an und die aufgeklärte Varianz liegt nun deutlich höher als im vorigen Modell bei 12 %. Auch Abb. 3 mit durchschnittlichen Marginaleffekten weist in diese Richtung. Dokumentiert wird hier der stärkere, zugleich nach dem breiteren Konfidenzintervall weniger sichere Einfluss. Die Resultate bestätigen *H 2*. Demnach ist ein diffuses Unbehagen an Ungleichheit in Deutschland viel wichtiger als Detailwissen zur Spitzensteuer.

Modell 3 nimmt die Einschätzung der eigenen Einkommensposition auf. Je höher die eigene Einkommensposition wahrgenommen wird, desto weniger wird die Vermögensteuer unterstützt. Da die Selbsteinstufung und das faktische Haushaltseinkommen korrelieren (Pearsons R = 0,4***), nimmt die Präsenz des Perzeptionsitems die durch das Einkommen erklärbare Varianz auf; der Koeffizient des Nettohaushaltseinkommens wird kleiner. Die Wahrnehmung der eigenen Position in der Einkommenshierarchie erklärt die Unterstützung für das verteilungspolitische Instrument „Vermögensteuer“ griffiger als das faktische Einkommen. Die Information zum Haushaltseinkommen gab Erklärungskraft bereits in Modell 2 an die Kritik an gesellschaftlicher Ungleichheit ab, in Modell 3 nochmals an die Perzeptionsvariable. Die durchschnittlichen Marginaleffekte (siehe Abb. 3) bestätigen dies. Der Koeffizient scheint sehr gering, aber wenn man ihn multipliziert mit dem Medianwert von 49 %, sinkt die Zustimmung um 0,35 Punkte auf der Skala, bei Haushalten mit noch höherer Selbstpositionierung, die 70 % der Bevölkerung unter dem eigenen Einkommen sehen, sinkt die Unterstützung schon um knapp einen halben Skalenpunkt. Auch das ist immer noch wenig, aber das Konfidenzintervall ist eng und das Ergebnis sicher: Wer die eigene Position als günstiger als die der übrigen Deutschen einschätzt, ist eher gegen die Vermögensteuer. Die subjektive eigene Position ist im Vergleich aber viel weniger einflussreich als die Unzufriedenheit mit der gesellschaftlichen Ungleichheit. Dass politische Einstellungen stärker durch den Blick auf das Ganze beeinflusst werden als durch die eigene Position, beobachten auch Studien zu „economic voting“: Die Parteienwahl wird stärker durch die Einschätzung der nationalen ökonomischen Lage als durch die geschätzte eigene Wirtschaftslage bestimmt (Lewis-Beck und Stegmaier 2007; Brenke und Kritikos 2017).

Den Einfluss der Mediennutzung auf die Unterstützung der Vermögensteuer prüft Modell 4 mit einer Dummy-Variablen für das intensive Nutzen von Fernsehen, Zeitungen und Internet mit dem Ziel politischer Information. Die Variable bildet die Intensität der Medienrezeption ab, ohne für im nächsten Schritt betrachtete Differenzen sensibel zu sein. Sie erzielt einen positiven, höchst signifikanten Koeffizienten. Die Unterstützung für die Vermögensteuer wächst offenbar mit intensiver Mediennutzung. Zugleich wird der Dummy für Realschulbildung im Kontrast zu höheren Abschlüssen insignifikant. Die intensive Mediennutzung kompensiert den negativen Effekt, den hohe Schulabschlüsse sonst üblicherweise für die Sympathie gegenüber Umverteilungspolitik bedeuten.

Da die abhängige Variable „Zustimmung zur Vermögensteuer“ linksschief verteilt ist, was die Voraussetzungen der OLS-Regression verletzt, wurde die Robustheit der Ergebnisse durch logistische Regressionsmodelle geprüft. Die Resultate sind strukturell gleich zur OLS-Regression: Alle Koeffizienten haben die gleichen Vorzeichen wie mit OLS, die Richtung der Effekte ändert sich nicht. Lediglich die Signifikanz der Variablen „Fehleinschätzung Spitzensteuer“ und „Perzeption eigene Position“ sinkt von höchst zu hoch signifikant im Fall der logistischen Regression und die erklärte Varianz (Modellgüte) wird geringer.

Die Zustimmung zur Vermögensteuer differiert nach der Parteiidentifikation: Fast alle Anhänger der Die Linke wollen sie (91 %), drei Viertel der Anhänger von Bündnis90/Die Grünen (76 %) und der SPD-Anhänger (74 %), 58 % der Anhänger der CDU/CSU und ähnlich 55 % der AFD-Anhänger. Nur die Anhänger der FDP unterstützen sie wenig (44 %). Die teils deutlichen Parteiunterschiede ergeben große Effekte in der multivariaten Regressionsanalyse. Das R‑Quadrat steigt von 13,9 % auf 20,3 %. Aber die signifikanten Effekte der Wahrnehmungsitems (Schätzung Spitzensteuer, eigene Position, Ungleichheit zu groß) bleiben. Da sich die Fallzahl halbieren würde auf 717 Befragte – die anderen gaben keine Parteiidentifikation an – und der Einfluss von Parteien auf Einstellungen zu „policy issues“ gut erforscht ist (z. B. Slothuus und Freese 2010; Slothuus und Bisgaard 2020), verzichte ich auf die Analyse dieses Merkmals.

### Frames in den Medien

**Figure 2:**
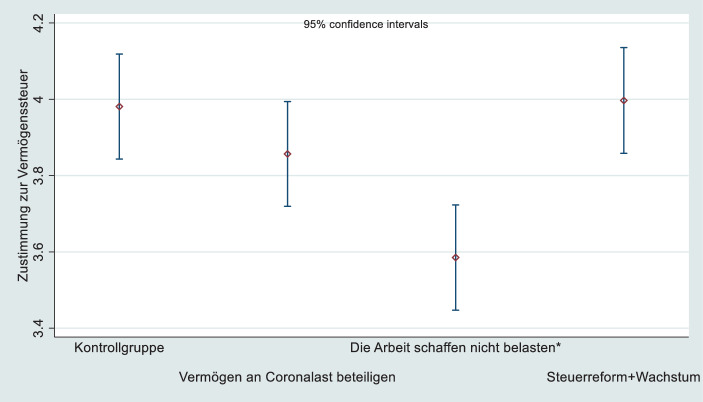

Erreichen Medieninhalte, die Parteien, Interessengruppen und Redaktionen in Umlauf bringen, die Wähler und Wählerinnen? Bewegen Rahmen die Einstellungen der Befragten in Richtung der jeweiligen Positionen zur Vermögensteuer? Abbildung 2 zeigt zunächst, ob sich die Unterstützung für Vermögensteuern durch die Treatments verändert. Wenn die Treatmentgruppen andere Durchschnittswerte bei der abhängigen Variable als die Kontrollgruppe aufweisen, lässt dies auf einen Effekt schließen. Nur ein Frame erzielt ein signifikant anderes Antwortverhalten als das der Kontrollgruppe. Das Treatment zum Risiko des Arbeitsplatzabbaus durch eine Besteuerung von Vermögen senkt die Unterstützung im Vergleich zur Kontrollgruppe höchst signifikant (*p* ≤ 0,001). Die Argumente zu einer Vermögensteuer, die die wegen Corona angehäuften Staatsschulden abbauen hilft oder in Infrastruktur und technologischen Wandel zu investieren sei, bewegt den Wert nicht signifikant über den der Kontrollgruppe. Die Kontrollgruppe hat diese Argumente, wie vermutet, bereits aufgenommen. Auch dürfte die langfristige mediale Berichterstattung über die Einkommens- und Vermögenszugewinne vor allem bei den Wohlhabenden (Scholtz 2010; Anselmann und Krämer 2012) das Argument popularisiert haben, dass diese Gruppen belastbar sind. Berichte zu den Finanzmarkt- oder Immobiliengewinnen und den Erbschaften des obersten 1 % der Bevölkerung oder breit wahrgenommene Publikationen wie *Das Kapital im 21. Jahrhundert* (Piketty 2015) machen die Besteuerung der „Reichen“ populär. Gleichwohl wären diese Vermutungen empirisch zu prüfen.

**Figure 3:**
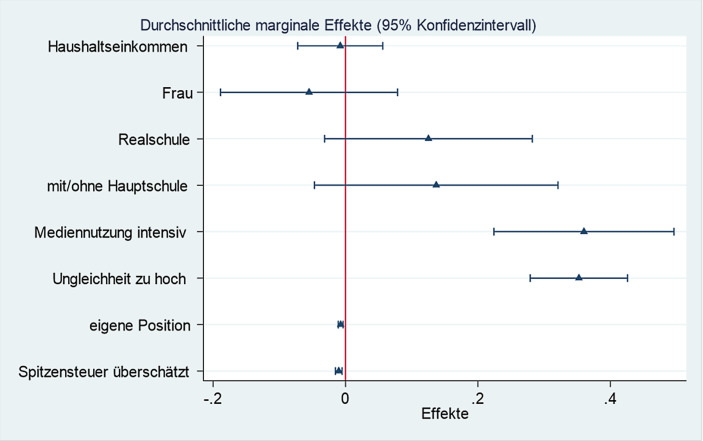

Die weitgehend fehlenden Reaktionen auf zwei der Treatments legen zum einen bereits in der öffentlichen Meinung angekommene Argumente pro Vermögensteuer nahe. Darüber hinaus spricht der sehr effektive Arbeitsplatzframe aber für die Benennungsmacht des „Widerlagers“, das die öffentliche Meinung an negative Anreize erinnert (siehe Abschn. 2.1). Wenn keine Arbeitsplatzsorgen aktiviert werden, erhalten die Rahmen der Befürworter der Vermögensteuer weitgehend Zustimmung. Wenn aber verfügbar gemacht wird, dass man „die Kräfte nicht belasten dürfe, die Arbeitsplätze schaffen“ erodiert der Konsens. Dies entspricht in hohem Maße der einleitend skizzierten Konstellation: Erst der Aufbau einer Drohkulisse über „investitionsmüde“ Unternehmer bremst den in Demokratien naheliegenden Anreiz, die Reichen zu belasten (Streeck 2013). Ob der Effekt des Frames, der Arbeitsplatzsorgen aktiviert, noch deutlicher ausfallen würde, wenn die empirisch im Sample der Onlinestudie unterrepräsentierte Gruppe mit geringer Schulbildung stärker vertreten wäre, ist offen. Denn in dieser Gruppe mögen die Sorgen um Arbeitsplätze schwerer wiegen als der Wunsch nach höheren Reichensteuern.

## Fazit

Der Beitrag greift die Debatte um den Einfluss ungenauer Wahrnehmungen ökonomischer Ungleichheit auf die Formung umverteilungspolitischer Präferenzen auf und prüft, inwieweit ein Bias auch die Unterstützung für die Vermögensteuer prägt. Zudem geht der Beitrag dem Einfluss der Medienberichterstattung auf perzipierte Ungleichheit nach, ein wenig untersuchtes und durch die Forschung zu (sozial-)politischen Einstellungen vernachlässigtes Thema.

Zunächst scheinen Vermögensteuern in Demokratien einfach machbar: Die Wählermehrheit entscheidet über Steuern, die eine Minderheit der Wohlhabenden belasten. Allerdings befindet man sich in einem dynamischen Feld, in dem ein Kampf um Benennungsmacht ausgetragen wird (Bourdieu 1985) und Akteure ihre jeweiligen Deutungen versuchen durchzusetzen. Wie Scheve und Stasavage (2016) betonen, hängt die Einführung von „Reichensteuern“ nicht von besonders großer Vermögensungleichheit, sondern von gesellschaftlichen Diskursen zur Privilegierung der Reichen einerseits und der Überlastung der Reichen andererseits ab. Eine Seite der um Benennungsmacht ringenden Akteure – die wirtschaftlichen und die liberal-konservativen politischen Eliten – inszeniert den essenziellen gesellschaftlichen Beitrag der Wirtschaft und erinnert an fehlende Arbeitsplätze, falls gute Bedingungen für Unternehmen fehlen. Die andere Seite erinnert an seit Jahren davonziehende Topeinkommen und Vermögen, was deren Beitrag zum Abbau von Ungleichheit und Staatsschulden rechtfertige. Die Medien machen die kritische Darstellung Wohlhabender ebenso verfügbar wie Argumente dazu, dass die Belastung Vermögender negativ auf Arbeitsplätze wirkt. Wie kommen die konkurrierenden Deutungen bei der Bevölkerung an?

Wegen der These einer in Deutschland nicht nur ungenau, sondern übersteigert perzipierten ökonomischen Ungleichheit mit infolgedessen hoher umverteilungspolitischer Nachfrage wurde geprüft, ob die steuerliche Belastung Reicher verzerrt wahrgenommen wird und wie dies die Unterstützung für Vermögensteuern beeinflusst. Die Daten des Onlinesurveys dokumentieren breite Unterstützung für Vermögensteuern, die aber sinkt, wenn der Spitzensteuersatz für Gutverdiener zu hoch eingeschätzt wird. Da Befragte die Spitzensteuer öfter über- als unterschätzen, dämpft der dominierende Bias die Sympathien zugunsten von Vermögensteuern. Die Wahrnehmung ist keineswegs in einer Weise verzerrt, die die verteilungspolitische Nachfrage anfacht. Insgesamt schätzen Befragte die steuerliche Belastung Reicher relativ realistisch ein. Dies dürfte damit zusammenhängen, dass die Spitzensteuer in den letzten Jahren oft ein Gegenstand der medialen Berichterstattung war. Die in dieser Studie dennoch empirisch nachgewiesene Tendenz der Bürger, die gegebene Steuerbelastung an der Spitze der Einkommen höher als sie faktisch ist wahrzunehmen, bremst eine politische Nachfrage, die Reiche weiter mit Steuern belastet, also ab.

Sehr viel mehr wird aber die Unterstützung für eine Vermögensteuer vom Urteil der Befragten beeinflusst, die Ungleichheit in der Gesellschaft sei zu groß. Eine Medienstrategie, die generell das Bild einer ungleichen und ungerechten Gesellschaft zeichnet, dürfte folglich „effektiver“ sein als Bürger besser über Steuersätze zu informieren. Auch die geschätzte eigene Lage erzielt einen höchst signifikanten Einfluss: Je höher Befragte ihre Position in der Einkommenshierarchie wahrnehmen, desto stärker lehnen sie eine Vermögensteuer ab. Aber im Vergleich zum Urteil über die gesamte Gesellschaft prägt die Einschätzung der eigenen Position die Unterstützung einer Vermögensteuer sehr viel weniger.

Die medienvermittelte politische Kommunikation ist eine wichtige Komponente der Präferenzbildung. Drei Framingexperimente testeten in den Medien gängige Rahmen zur Vermögensteuer. Die beiden Experimente mit positiven Aspekten der Vermögensteuer – Beitrag zum Schuldenabbau und zu Zukunftsinvestitionen – können die bereits in der Kontrollgruppe hohe Unterstützung nicht weiter steigern. Diese Frames sind durch langjährige Debatten um „Reichensteuern“ bereits in der öffentlichen Meinung verankert. Aber Interessengruppen der Wirtschaft und wirtschaftsnahe Parteien bringen einen Frame erfolgreich ins Ringen um Deutungsmacht ein: Das Argument, dass man „Kräfte, die Arbeit schaffen“ nicht zu sehr belasten dürfe, da sie sonst abwandern, dämpft höchst signifikant den Konsens zugunsten der Vermögensteuer.

Die Benennungsmacht beider Seiten ist Ergebnis langfristiger Medienarbeit, die eine Querschnittstudie wie diese nicht fassen kann. Künftige Forschung sollte analysieren, wie Medieninhalte langfristig einerseits die Privilegierung der Reichen und andererseits die Überlastung der Wirtschaft in Szene setzten und welche Seite in den vergangenen Jahren in den Medien dominierte.

Trotz wiederholter Anläufe verschiedener Parteien gibt es in Deutschland keine Vermögensteuer und ihre Einführung ist fraglich. Man kann dies als mangelnde Responsivität der Politik diskutieren. Aber ebenso spielt die Dynamik der Präferenzformation eine Rolle: Wie ich zeigte, senkt das Framing mit bedrohten Arbeitsplätzen die Zustimmung zur Vermögenssteuer fast auf den Skalenmittelwert, der als „teils/teils“ beschriftet ist, also Unbestimmtheit indiziert. Bei Verfügbarkeit von Kontraargumenten ist die Unterstützung keineswegs so positiv wie es durch eine einfache Surveyfrage scheint. Experimente bilden diverse Positionen im „Kampf um Benennungsmacht“ besser ab.

Zudem ist im Survey geäußerte Zustimmung keine Wahlentscheidung für Parteien, die Vermögensteuern umsetzen, da die Parteienwahl auch nach anderen Themen (z. B. Flüchtlings- oder Umweltpolitik) getroffen wird.^24^ Wenn die Einstellungsforschung dem relativen Gewicht anderer Issues im Vergleich zu verteilungspolitischen Themen mehr Aufmerksamkeit schenken würde, wäre sie näher am realen Potenzial der Unterstützung für Umverteilung.

## Anhang

**Table 2:**

	In %
Lehne voll und ganz ab	7,9
Lehne eher ab	8,5
Teils/teils	17,6
Unterstütze eher	25,6
Unterstütze voll und ganz	40,5

Daten: Onlinesurvey Vermögensteuer 2020. Gewichtet. *N* = 1260

**Table 3:**

	Eigenes Sample	ALLBUS 2018	Destatis/ESS
*Schulabschluss, in %*			Destatis
Haupt‑/Volkschule/o. Abs	25,3	25,1	32,6
Realschule	43,7	34,8	30
Abitur/FHreife	31	40,1	33,5
*Haushaltsnettoeinkommen, in %*			ESS^a^
1) unter 1000 €	13,3	16,2	6,7^a^
2) 1000 bis < 2000 €	17	18,7	15,5^a^
3) 2000 bis < 4000 €	43	39,9	40,6^a^
4) 4000 bis < 5000 €	15	11,1	23,4^a^
5) 5000 € und mehr	11,7	13,6	13,9^a^
*Alter, in %*			ESS
18–29 J	20	15,2	15,8
30–59 J	55	56,6	54,8
60–75 J	25	28	29,4
*Geschlecht*			
Weiblich	50	49	
*Erwerbsstatus, in %*		^b^	
Erwerbstätig	60,2	54,9	Destatis und ESS nur Erwerbsalter
Rentner/Pensionär	22,9		
Nicht erwerbstätig	15,2	38,5	
Schüler	1,7		

^a^Das ESS/Welle 9 verwendet zehn Einkommensgruppen, die etwas anders als die Gruppen des eigenen Onlinesamples abgegrenzt sind. Identische Gruppen lassen sich nicht bilden, aber eine Annäherung ist möglich. Nach den Abgrenzungen des ESS ergeben sich: 1) unter 1140 €; 2) 1141–1950 €; 3) 1951–3750 €; 4) 3751–5670 €; 5) 5671 € und mehr

^b^Die Frage nach der Berufstätigkeit im ALLBUS hat nur die Antwortkategorien Berufstätigkeit ganztags, halbtags oder nebenher und nichterwerbstätig, bezieht sich aber auch auf über 65-Jährige

**Table 4:**

	Kontrollgruppe	Coronaframe	Arbeitsplatzframe	Wachstum + Steuerreform	Gesamt
*Schulabschluss*					
Volksschule/oA	26,8	24,2	28,1	22,0	25,3
Realschule	41,2	42,1	43,2	48,4	43,7
Abitur/FHreife	32	33,6	28,7	29,5	31
*Haushaltsnettoeinkommen*					
Unter 2000 €	27	24,8	29,2	27,1	27
2000 bis < 4000 €	41,2	39,4	43,3	36	40
4000 € und mehr*	31,8	35,9	27,4	36,9	33
*Alter*					
18–29 J	18,7	21	20,5	19,7	20
30–59 J	56,4	54,2	55,4	54	55
60–75 J	24,9	24,9	24,1	26,3	25
*Geschlecht*					
Weiblich	51,4	49,6	50,2	48,9	50

Anteil Bundesländer: West 75,3 %, Ost 25,7 %

## Footnotes

1 Zur Rolle von Interessengruppen in den USA, die Kampagnen finanzieren, um die öffentliche Meinung zur Einkommensungleichheit zu lenken, siehe Hacker und Pierson (2010).

2 https://www.bertelsmann-stiftung.de/de/presse/pressemitteilungen/pressemitteilung/pid/umfrage-bevoelkerung-sieht-soziale-schieflage-in-deutschland (Zugegriffen: 20. Mai 2020).

3 https://www.tagesschau.de/inland/deutschlandtrend-1897.html (Zugegriffen: 20. Mai.2020).

4 Streecks Analyse zum „Investitionsstreik“ der Unternehmen in den 1980er- und 1990er-Jahren ist ein Beispiel für wahrgemachte „inverse Effekte“ mit der Folge steigender Arbeitslosigkeit (Streeck 2013, S. 45).

5 Wenn Einkommen von Berufen an der Spitze der Berufshierarchie geringer, als sie faktisch sind, geschätzt werden, Löhne am unteren Ende für einfache Arbeiter aber eher überschätzt werden, hat das einen stauchenden Effekt (Kelley und Evans 1993; Osberg und Smeeding 2006). Ob diese schon älteren Befunde aktuell noch gelten, wäre eingehender zu prüfen. Nach neueren Daten werden Spitzeneinkommen überschätzt, die Löhne am unteren Ende der Einkommensskala hingegen unterschätzt (Pontusson et al. 2020, S. 10). Ein Einfluss der Medienberichterstattung liegt auch hier nahe.

6 Bei dem Befund, dass Befragte unterschätzen, wie hoch die von Einkommensmillionären gezahlten Steuern sind (Niehues 2019, S. 92), muss an den Satz von Eriksson und Simpson (2012, S. 741) erinnert werden: „What do Americans know about inequality: It depends on how you ask them.“ Die Darstellung numerischer Größen beeinflusst, was Befragte schätzen.

7 Demgegenüber widmen sich mehrere Arbeiten den Medieninhalten zu ökonomischer Ungleichheit (Schröder und Vietze 2015; Bank 2017) oder zu Vermögensteuern (Lichtenstein et al. 2016; Theine und Grisold 2020), ohne die Wirkung auf die öffentliche Meinung zu prüfen.

8 Die Sekundärdaten von Mediatenor haben den Vorteil, dass sie auch Nachrichten in ARD und ZDF und die Bildzeitung (neben Focus und Spiegel) berücksichtigen. Diese schwerer langfristig zugänglichen Medientypen fehlen in den meisten Medieninhaltsanalysen, die sich auf Archive der deutschen Qualitätszeitungen stützen.

9 Zu Valenzen und deren Einfluss allerdings auf Einstellungen zur Europäischen Integration siehe Schuck und de Vreese (2006) sowie Vliegenthart et al. (2008).

10 BT-Drucksache 17/10778. Gesetzvorschlag Die Linke: Reichtum UmFairteilen.

11 BT-Drucksache 17/10770. Gesetz zur Erhebung einer Vermögensabgabe.

12 Umfrage zu „Gerechtigkeit in Deutschland: Einstellungen der Bevölkerung zu Fragen der Verteilungsgerechtigkeit und Steuerpolitik“. Deutscher Paritätischer Wohlfahrtsverband (2013).

13 DGB Bundesvorstand (2019).

14 Instrumente gegen die Krise. Ergebnisse einer Repräsentativbefragung in Deutschland. Oktober 2012.

15 https://www.spd.de/aktuelles/vermoegensteuer/.

16 https://www.spd.de/standpunkte/steuern/.

17 https://indieneuezeit.spd.de/fileadmin/pv/Dokumente/BPT2019/Beschluesse/B11_Die_Vermoegensteuer_wieder_einfuehren_Verteilungsgerechtigkeit_herstellen.pdf.

18 Siehe Audio https://www.ardaudiothek.de/tagesschau/03-11-2020-tagesschau-20-00-uhr/82658120; oder Video: https://www.ardmediathek.de/daserste/video/tagesschau/tagesschau-20-00-uhr/das-erste/Y3JpZDovL2Rhc2Vyc3RlLmRlL3RhZ2Vzc2NoYXUvNjNkMDU0YTAtMTFiMC00OWM0LTljMWItN2I0NzFlYjIwOTJi/.

19 30 Personen schätzten den Spitzensteuersatz auf über 80 %. Diese Fälle werden ausgeschlossen.

20 „Es gibt unterschiedliche Quellen, aus denen man sich über Politik informieren kann. Woher bekommen Sie aktuell die meisten Informationen über Politik?“ Die Optionen Fernsehen, Internet, Radio, Zeitung (auch Internetausgabe), persönliche Gespräche, soziale Medien, wie Facebook und Twitter, können je einzeln dichotom (ja/nein) mehrfach gewählt werden.

21 Antwortoptionen „täglich“, „mehrmals die Woche“, „mehrmals im Monat“, „mind. 1‑mal pro Monat“, „seltener“, „nie“.

22 Zum Beispiel: Clemens Fuest: Kosten und Nutzen einer deutschen Vermögensteuer. Frankfurter Allgemeine Zeitung 04.10.2019 (Wirtschaft, S. 18) und Olaf Scholz: „Viele Löhne sind eine Beleidigung.“ Interview geführt und veröffentlicht durch die Frankfurter Allgemeine Zeitung 31.10.2020 (Wirtschaft S. 18), aber exemplarisch für die aktuelle sozialdemokratische Position.

23 Nach einer Analyse mit dem German Internet Panel (Welle 51, Jan. 2021, ZA7759) nutzten nur 60 % nicht das Internet, um Wissensfragen dort zur Europäischen Union zu beantworten (z. B. „Aus wie vielen Abgeordneten besteht das Europäische Parlament derzeit?“). In den eigenen Daten rechne ich mit weniger „Googeln“, da zum Schätzen aufgefordert wurde und weniger fremde Detailfragen gestellt wurden als im German Internet Panel. Stellt man einen gewissen Umfang an Informationsbeschaffung per Internet in Rechnung, wäre im Grunde von mehr Fehleinschätzungen bei der Spitzensteuer auszugehen. Da die Informationssuche im Internet sowohl von Befragten mit über- als auch unterschätzten Werten praktiziert werden dürfte, ist sie letztlich unschädlich und verändert keine Resultate.

24 Das zeigt sich etwa an der hohen Zustimmung zur Vermögensteuer, aber der nicht gleichermaßen hohen Wahlabsicht der SPD, obwohl sie die Einführung einer Vermögensteuer auf ihrem Parteitag im Dezember 2019 beschloss. Siehe auch https://www.tagesschau.de/inland/deutschlandtrend-1897.html.
